# An unusual cause of adult onset cerebellar ataxia with hypogonadism

**DOI:** 10.4103/0972-2327.48852

**Published:** 2009

**Authors:** Ramshekhar N. Menon, Nirav Sanghani, Mahendra Javali, Neeraj Jain, Arun B. Shah

**Affiliations:** Department of Neurology, BYL Nair Ch. Hospital and TN Medical College, Mumbai Central, Mumbai - 400008, Maharashtra, India

**Keywords:** Celiac disease, gluten ataxia, hypogonadism

## Abstract

We report an unusual case of sporadic adult onset cerebellar ataxia with hypogonadism. A 40-year-old unmarried man presented with progressive ataxia and dysarthria along with complaints of non-development of secondary sexual characteristics and erectile dysfunction. There were complaints of intermittent diarrhea. Clinical examination revealed a pan-cerebellar syndrome with features of hypoandrogenism. No eye movement abnormalities were evident. There were signs of malabsorption. Investigations confirmed the presence of auto-antibodies found in celiac disease, and a duodenal biopsy confirmed the same. Hypoandrogenism was postulated to be due to hypergonadotropic hypogonadism which has been mentioned in a few patients of celiac disease. However, the pattern seen in our patient was of a hypogonadotropic hypogonadism. This is probably secondary to an autoimmune hypophysitis seen in some patients in the absence of other clinical manifestations. Autoantibody testing should be a diagnostic necessity in any adult with a sporadic cerebellar ataxia.

## Introduction

An adult presenting with a sporadic chronic progressive cerebellar ataxia offers the physician a myriad of diagnostic possibilities. Celiac disease as the cause of ataxia is a known entity with various clinical features that would lead one to dwell into its consideration.

It has been estimated that 10% of patients with celiac disease develop neurological complications.[1] For every one patient with celiac disease who presents with gastrointestinal complaints, there are seven patients with celiac disease who have no gastrointestinal symptoms.[2] Neurological disorders associated with celiac disease were recognized as early as 1908 with majority of the case reports documenting either a peripheral polyneuropathy or a sensory ataxia. Cerebellar dysfunction with focal loss of Purkinje cells and neuronal degeneration in the dentate nuclei was recognized in a minority of patients.[3] It has been shown that the deficiency of folic acid, tocopherol, vitamin B12 played no significant roles in precipitating the neurological manifestations.[3,4] Postulates on additional biopterin and carnitine deficiency states in celiacs exist.[4] The association of gluten in conditions such as depression, brain-stem encephalitis, myoclonic epilepsy, leukoencephalopathy has also been described.[4] Even in children, the variability of neurological disorders that occur in celiac disease is broader than previously reported and includes “softer” and more common neurologic disorders such as chronic headache, developmental delay, hypotonia, and learning disorders or ADHD.[5] Mucosal pathology does not represent an obligatory condition of ataxia with gluten sensitivity.

Ataxia in a patient with hypogonadism represents a wide differential. The familial syndromes usually predominate. The most well known entity is the Gordon-Holme's spinocerebellar ataxia syndrome (GHS). Other syndromes include spinocerebellar hereditary degeneration (SCHD) associated with the Klinefelter syndrome, Boucher-Neuhauser syndrome and Richards-Rundle syndrome. In celiac disease, endocrinopathy can manifest as hypogonadism.

We would like to report one such unique case of this so called “gluten ataxia” who was additionally detected with hypogonadism as a prominent feature.

## Case Report

The patient was a 40-year-old unmarried male who was symptomatic since last 7 years for progressive ataxia. He also complained of clumsiness in fine activities including writing. There was a history of slurring of speech for the past 2 years. In addition, he had also noted a paucity of facial, axillary and pubic hair and impotence including absence of early morning erections. Many indigenous medications were consumed for the same. Past history was significant for bouts of diarrhea since his early twenties.

### Examination

**Figure 1:**
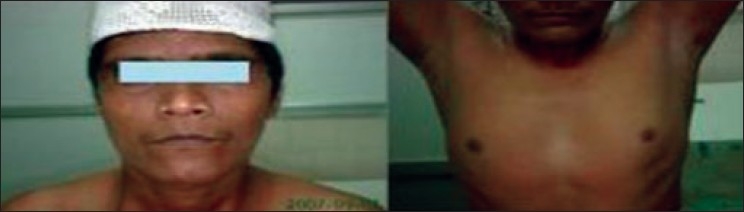
Features of hypoandrogenism

Clinical examination revealed a well nourished, moderately built adult with a BMI of 21 kg/sq.m. His arm span was 182 cm with a height of 170 cm. Hypoandrogenism was seen with reduced facial, axillary and pubic hair [Figure 1]. Gynecomastia was present. General physical examination was normal. Higher mental functions were normal with an MMSE of 30/30. Speech revealed scanning dysarthria. Cranial nerve examination revealed bilateral gaze evoked nystagmus with fast phase in direction of gaze. Saccades and pursuit were normal. Fundus examination was unremarkable. No pyramidal or sensory findings were noted. Romberg's test was negative. Ankle reflexes were bilaterally hypoactive. Bilaterally symmetric dysmetria, dyssynergia, incoordination, and truncal ataxia were noted. Stance was broad based with gait ataxia. In summary, this man had a progressive pan-cerebellar syndrome with evidence of hypogonadism. In view of the history of recurrent diarrhea, a possibility of gluten ataxia was considered.

### Investigations

**Figure 2:**
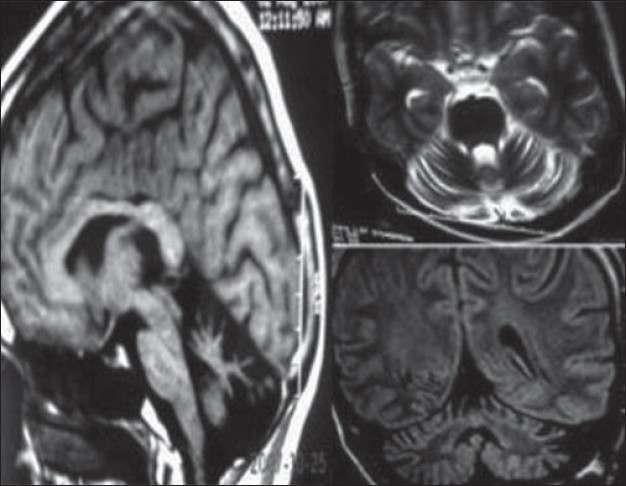
MRI images – isolated cerebellar atrophy

**Figure 3:**
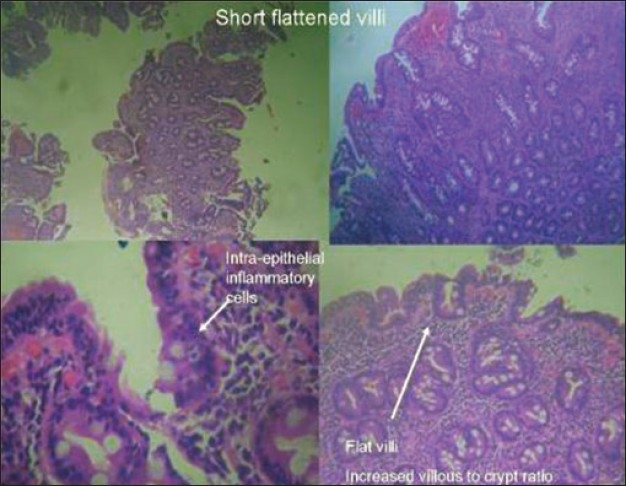
Histological characteristics on duodenal biopsy of the patient

Hb = 11.3 gm% with a normocytic normochromic picture. Total leukocyte counts = 8,700/ cu.mm; platelets adequate. Fasting blood sugar level = 84 mg/dl; 2 hour post lunch sugar = 106 mg/dl. Blood urea nitrogen = 13 mg/dl; serum creatinine = 0.6 mg/dl; sodium = 142 mEq/L; potassium = 4.1 mEq/L Serum calcium = 8.9 mg/dl; phosphorus = 4.2 mg/dl. Total proteins = 7.5 g/dl; serum albumin level = 3.9 g/dl. AST levels = 22 IU; ALT levels= 23 IU; Alkaline phosphatase = 110 IU. Total cholesterol = 119 mg%; serum triglyceride= 206 mg%. HIV ELISA and other viral markers were negative. Stool routine and microscopy revealed minimal fat globules; no occult blood or parasites. Serum vitamin B12 levels = 795.7pg/ml; vitamin E levels = 7.60 mg/L (6–19 mg/L). Serum FSH levels = 1.11 mIU/ml (1.4–18.1 mIU/ml); LH levels = 0.30 mIU/ml (1.5–9.3 mIU/ml). Serum testosterone levels = 0.33 ng/ml (2.7–10.7 ng/ml) T3=125 ng/dl; T4= 9.5 μg/dl; TSH=4.46 μU/ml. Testicular volume – Right = 9 cc and Left = 10.5 cc (normal = 13–19 cc). Nerve conduction studies revealed bilaterally attenuated H reflexes with normal Sensory Nerve Action Potentials (SNAPs) and Compound motor action potentials (CMAPs). Magnetic Resonance Imaging (MRI) of the brain revealed gross cerebellar atrophy [Figure 2]. On gastroscopy there was mild gastric atrophy with mild duodenitis in the D1 segment. Histopathology of biopsy specimens from both areas revealed features which were consistent with celiac sprue (short flattened villi, intra-epithelial lymphocytic infiltrates, increased villous-crypt ratio) [Figure 3].

Serum IgA Anti-Gliadin Antibodies (AGA) = 31.2 U/ml (*n* = ≤11 U/ml) Serum IgG Anti-gliadin Antibodies= 17.4 U/ml (n = 11–17 U/ml). Serum IgA Anti-tissue Transglutaminase (TTG) = 168.90 U/ml (0–20 U/ml) Serum IgA Anti-endomysial Antibodies (EMA) = 243.40 U/ml (0–20 U/ml)

On the basis of the above findings, patient was diagnosed to have gluten ataxia and enteropathy with additional hypogonadotropic hypogonadism. He has been initiated on a gluten-free diet and is under evaluation for hormonal replacement therapy.

## Discussion

A patient with a sporadic, chronic progressive pan-cerebellar syndrome poses a challenge to the clinician. The investigations are directed to finding a treatable cause of ataxia. Gluten ataxia is an important and under-recognized cause, with possible benefits of dietary modification on neurological outcome.

Autoantibodies detected in patients of celiac disease are postulated to cause the neurological manifestations. Hadjivassiliou looked for IgG and IgA AGA in two groups of neurological patients. The first group of 53 patients had a wide range of idiopathic neurological dysfunction and the second group of 94 patients had specific neurological diagnoses. A higher prevalence of positive AGA was found in the first group compared with the second (57% vs 5%). A subset of patients from the first group was also shown to have histological evidence of celiac disease on gut biopsy. Gluten sensitivity was thus shown to also present solely with neurological dysfunction.[6] Ataxia was found to be the most common neurological manifestation. Autopsy revealed lymphocytic infiltration of cerebellum, posterior columns and peripheral nerves. The term “gluten ataxia” was thus proposed.[7]

Organ-specific manifestations can occur in isolation or in combination with one another. Only a proportion of patients presenting with neurological dysfunction associated with gluten sensitivity will also have an enteropathy.[8] The remaining patients have no histological evidence of small bowel involvement but have serological markers (serum AGA) in keeping with gluten sensitivity, a situation analogous to dermatitis herpetiformis. Genetic susceptibility in the form of the Human Leukocyte Antigen (HLA) typing may play an important role in this disease. HLA DQ2 is found in up to 90% of patients with celiac disease.

One of the largest cohorts of patients of gluten ataxia (68 patients) revealed a mean age at onset and duration of illness similar to our patient. Majority of the patients had ocular signs, dysarthria, appendicular and gait ataxia. Most had MRI features of cerebellar atrophy with only a small subset revealing white matter lesions. Gastrointestinal symptoms were seen in only a minority of the patients with gluten sensitivity, proven in only 24%. It was also seen that AGA were detected in a statistically significant number of patients of sporadic idiopathic cerebellar ataxias in comparison to familial ataxias, MSA-C and normal controls. Thus, AGA testing is essential at first presentation of patients with sporadic ataxia.[9]

In a study from Italy, patients with sporadic ataxia were found to be more likely to have celiac disease (three out of 24) than a group of patients with familial ataxia (zero out of 23), with celiacs positive for mainly IgG AGA.[10] Furthermore, the detection of anti-TTG and anti-EMA is highly predictive of the condition in comparison to AGA (seen in 10% of normal controls).[11] AGA have been described in patients with Huntington's and inherited ataxias[12–14] rendering a valid point for those who have reservations on the antibody mediated mechanism of neuronal injury. The estimation of additional antibodies as screening tests for gluten ataxia in patients with the diagnosis of cerebellar ataxia of unknown origin renders greater specificity to the diagnosis.[15] The titers may not necessarily correlate with neurological signs/symptoms or the diet followed.[16]

The fact that the disease is strongly associated with the same HLA haplotypes found in celiac disease not only demonstrates celiac disease and ataxia with gluten sensitivity to be part of the same disease entity but supports the hypothesis of an immunological pathogenesis of cerebellar degeneration.[17]

Reasonable evidence has been put forth to support immune-mediated cerebellar injury. Prevalence of central nervous system antineuronal antibodies was significantly higher in neurological than in other patients of celiac disease.[18] Hadjivassiliou *et al*. assessed the reactivity of sera from patients with gluten ataxia, newly diagnosed patients with celiac disease without neurologic dysfunction, patients with other causes of cerebellar degeneration, and healthy control subjects using indirect immunocytochemistry on human cerebellar and rat CNS tissue. At high dilutions (1:800), staining was seen only with sera from patients with gluten ataxia but not in control subjects, to suggest that gluten ataxia patients have antibodies against Purkinje cells.[19]

It has also been recently shown that anti-TTG IgA antibodies are present in the gut and brain of patients with gluten ataxia with or without an enteropathy in a similar fashion to patients with celiac disease, latent celiac disease, and dermatitis herpetiformis but not in ataxia control subjects. Widespread IgA deposition around vessels was found in the brain of the patient with gluten ataxia but not the control brain. The deposition was most pronounced in the cerebellum, pons, and medulla.[20]

Case reports are available as well regarding the effectiveness of gluten-free diet in such patients and hence the importance of arriving at the diagnosis cannot be underestimated. The evidence is however not compelling.[21] Concurrent nutritional deficiencies (B12, folate, pyridoxine, and calcium) should be corrected. There have been a few reports of immunosuppressive treatment in patients with celiac disease and neurological syndromes. This proved helpful in a case of vasculitis[22] and in a patient with relapsing-remitting brainstem cerebellar syndrome.[23] Intravenous steroids and immunoglobulins were not helpful in a case of progressive leukoencephalopathy.[24]

### Hypogonadism

The association of hypogonadism with celiac disease is quite intriguing. Multiple endocrine manifestations have been described in relation such as type I diabetes mellitus (2%–5% of celiacs) and thyroid disease. A similar association has been proposed with other autoimmune conditions such as primary biliary cirrhosis and Sjogren's Syndrome. The basis of this is unclear. Approximately 90% of celiac disease patients with concomitant endocrinopathies share the HLA DR3-HLA DQ2 configuration, and most of the remainder express the DR4-DQ8 haplotype. The coexistence of these diseases could also be explained by molecular mimicry, loss of immune tolerance and increased intestinal permeability that facilitates external antigens such as food proteins, bacterial products, and endotoxins to enter the intestinal lamina propria, thus leading to the activation of autoimmune phenomena.[25] Gluten in itself, viruses, selenium deficiency and cigarette smoking are also thought to contribute.

Hypogonadism in celiacs contributes to growth failure and infertility. Predominant peripheral rather than central forms have been described. Farthing *et al*. found that the most striking endocrine findings in untreated celiacs were increased plasma testosterone and free testosterone index, reduced dihydrotestosterone (testosterone's potent peripheral metabolite) and raised serum LH, a pattern of abnormalities indicative of androgen resistance. As jejunal morphology improved, hormone levels appeared to return to normal.[26]

Symptoms of celiac disease may be confusingly similar to those in concomitant autoimmune hypophysitis, which again may give rise to diagnostic delay of either condition. Patients in the pediatric age group with atypical celiac disease present with short stature and iron deficiency anemia.[27] P. Collins *et al*. described three celiac patients who also were found to be suffering from hypopituitarism.[28] Manifestations were recurrent hypoglycemic events, asthenia and growth failure respectively. These symptoms were initially attributed to poor celiac control, but were later found to be caused by concomitant hypopituitarism. It might have been of autoimmune origin; at least none of the subjects had a pituitary mass. This could probably explain the coexistence of hypogonadotropic hypogonadism in our patient.

Literature did not reveal any such case with the combination of clinical findings of gluten ataxia with hypogonadotropic hypogonadism. The importance of diagnosis of this condition cannot be overemphasized as clinical response to gluten-free diet is well documented, though not conclusive. The absence of distinctive neurological features in patients of gluten ataxia suggests that a search should be made for auto-antibodies in all ataxic patients without a definite diagnosis.
